# Contribution of Resting Conductance, GABA_A_-Receptor Mediated Miniature Synaptic Currents and Neurosteroid to Chloride Homeostasis in Central Neurons

**DOI:** 10.1523/ENEURO.0019-17.2017

**Published:** 2017-03-23

**Authors:** Tushar D. Yelhekar, Michael Druzin, Staffan Johansson

**Affiliations:** Department of Integrative Medical Biology, Umeå University, Umeå, SE-901 87, Sweden

**Keywords:** chloride homeostasis, GABA_A_ receptor, KCC2, miniature postsynaptic current, neurosteroid, resting chloride conductance

## Abstract

Maintenance of a low intraneuronal Cl^–^ concentration, [Cl^–^]_i_, is critical for inhibition in the CNS. Here, the contribution of passive, conductive Cl^–^ flux to recovery of [Cl^–^]_i_ after a high load was analyzed in mature central neurons from rat. A novel method for quantifying the resting Cl^–^ conductance, important for [Cl^–^]_i_ recovery, was developed and the possible contribution of GABA_A_ and glycine receptors and of ClC-2 channels to this conductance was analyzed. The hypothesis that spontaneous, action potential-independent release of GABA is important for [Cl^–^]_i_ recovery was tested. [Cl^–^]_i_ was examined by gramicidin-perforated patch recordings in medial preoptic neurons. Cells were loaded with Cl^–^ by combining GABA or glycine application with a depolarized voltage, and the time course of [Cl^–^]_i_ was followed by measurements of the Cl^–^ equilibrium potential_,_ as obtained from the current recorded during voltage ramps combined with GABA or glycine application. The results show that passive Cl^–^ flux contributes significantly, in the same order of magnitude as does K^+^-Cl^–^ cotransporter 2 (KCC2), to [Cl^–^]_i_ recovery and that Cl^–^ conductance accounts for ∼ 6% of the total resting conductance. A major fraction of this resting Cl^–^ conductance is picrotoxin (PTX)-sensitive and likely due to open GABA_A_ receptors, but ClC-2 channels do not contribute. The results also show that when the decay of GABA_A_ receptor-mediated miniature postsynaptic currents (minis) is slowed by the neurosteroid allopregnanolone, such minis may significantly quicken [Cl^–^]_i_ recovery, suggesting a possible steroid-regulated role for minis in the control of Cl^–^ homeostasis.

## Significance Statement

The Cl^–^ concentration in central neurons is critical to normal synaptic function and if not properly regulated may contribute to epilepsy, chronic pain, and other pathologies. Here, we introduce a novel method to quantify the resting Cl^–^ conductance of the neuronal membrane and show how this conductance contributes to the recovery of Cl^–^ concentration after a high Cl^–^ load. We also clarify that ion channels of the GABA_A_ receptor type underlie a major fraction of the resting Cl^–^ conductance and show that an endogenous neurosteroid may quicken recovery of Cl^–^ concentration via effects of spontaneously released neurotransmitter on the GABA_A_ receptors. The findings are important for understanding the neuronal Cl^–^ homeostasis, critical to normal brain function.

## Introduction

The intraneuronal Cl^–^ concentration, [Cl^–^]_i_, is critical for synaptic communication. The inhibitory actions of GABA and glycine depend on a relatively low [Cl^–^]_i_. [Cl^–^]_i_ may, however, rise as a consequence of intense synaptic activity. When excitatory inputs depolarize the membrane, the driving force for inward Cl^–^ flux through channels opened by GABA or glycine increases and may impose a strong Cl^–^ load on the neuron. Cl^–^ flows into the cell also during shunting inhibition (Doyon et al., 2016b). In some cases, the raised [Cl^–^]_i_ changes the effect of GABA to excitatory (Khalilov et al., 2003; Astorga et al., 2015). Restoring [Cl^–^]_i_ after large perturbations may thus be expected crucial for normal inhibitory GABAergic synaptic function. The K^+^-Cl^–^ cotransporter 2 (KCC2) is critical for the control of [Cl^–^]_i_ and likely contributes significantly to [Cl^–^]_i_ restoration (Doyon et al., 2016b). However, as a consequence of the negative resting potential maintained by neurons, passive Cl^–^ flux through Cl^–^ conducting membrane pathways may also contribute to the recovery of a low [Cl^–^]_i_. In the absence of Cl^–^ transporters, the Cl^–^ equilibrium potential (*E*_Cl_) will settle at the membrane resting potential. The relative importance of KCC2 and passive, “conductive” flux is at present unknown.

Conductive [Cl^–^]_i_ recovery depends on the resting membrane Cl^–^ conductance (*g*_Cl_). There is, however, no generally accepted view on the resting Cl^–^ permeability or *g*_Cl_ in central neurons. A low resting permeability has been suggested by Thompson et al. (1988), but a significant conductance has been described in other studies (Forsythe and Redman, 1988; Sacchi et al., 1999; Müller, 2000).

The molecular basis for resting *g*_Cl_ is also not well understood. It has been proposed that ClC-2 channels are general contributors to the resting *g*_Cl_ in neurons (Rinke, 2010; but see Stölting et al., 2014). However, the expression of ClC-2 is highly restricted to specific neuronal types (Smith et al., 1995) or neuronal compartments (Földy et al., 2010). Another contribution to resting *g*_Cl_ may be provided by GABA_A_ receptors, which underlie a tonic conductance in some neurons (Semyanov et al., 2004; Belelli et al., 2009).

It seems possible that also the spontaneous GABA release that is seen as miniature postsynaptic potentials/currents (minis) contributes significantly to resting *g*_Cl_. The functional role of such minis is not clear. Although they imply relatively small currents, we hypothesized that they may be important for conductive recovery of [Cl^–^]_i_ after perturbations, at least when prolonged by endogenous neurosteroids that target GABA_A_ receptors and in some conditions also enhance GABA release (Haage and Johansson, 1999).

In the present work, we estimated the contribution of passive Cl^–^ flux to [Cl^–^]_i_ recovery, in preoptic neurons from rat. We also developed a novel method to estimate the relative *g*_Cl_ at rest and analyzed whether ClC-2 channels and GABA_A_ receptors contribute to resting *g*_Cl_. Finally, we tested the hypothesis that spontaneous GABA release is important for [Cl^–^]_i_ recovery. To improve the electrical recording conditions and to avoid possible [Cl^–^]_i_ redistribution between neuronal compartments, we used acutely isolated neurons from which neurites were largely removed, but which retain attached presynaptic nerve terminals that enable analysis of spontaneous GABA release (Haage et al., 1998). Cells were loaded with Cl^–^ by combining GABA or glycine application with a depolarized voltage and [Cl^–^]_i_ was obtained from measurements of *E*_Cl_ by gramicidin-perforated patch recordings with GABA or glycine application in combination with voltage ramps (Karlsson et al., 2011; Yelhekar et al., 2016).

Here, we show that passive Cl^–^ flux contributes significantly to [Cl^–^]_i_ recovery after a high load, and, using our novel method, we also show that Cl^–^ conductance accounts for ∼6% of the total resting conductance. A major fraction of this resting Cl^–^ conductance is picrotoxin (PTX)-sensitive and thus likely due to GABA_A_ receptors. Further, we show that when the decay of GABA_A_ receptor-mediated minis is slowed by the neurosteroid allopregnanolone, such minis contribute significantly to [Cl^–^]_i_ recovery, thus suggesting a possible steroid-regulated role for minis in the control of Cl^–^ homeostasis.

## Materials and Methods

### Ethical approval

All animal procedures were performed in accordance with the regulations of the regional ethics committee for animal research (Umeå djurförsöksetiska nämnd, approval number A9-14).

### Preparation of neurons

Acutely dissociated neurons were prepared from in total 148 young, male Sprague Dawley rats, 50-110 g, three to five weeks of age (Karlsson et al., 1997). The animals were housed with *ad libitum* access to food and water under a 12/12 h light/dark cycle. They were killed by decapitation without anesthetics, the brain removed, and 200- to 300-μm-thick coronal slices containing the medial preoptic area were cut. Individual neurons were isolated by application of a vibrating glass rod just above the slice, at the location of the medial preoptic nucleus. The major parts of neurites were thus removed, but functional presynaptic terminals remained attached to the postsynaptic cell bodies (Haage et al., 1998).

### Solutions used for preparation and for recording

A solution of the following composition was used to cut and incubate brain slices and for mechanical dissociation of individual neurons: 150 mM NaCl, 5.0 mM KCl, 2.0 mM CaCl_2_, 1.2 mM MgCl_2_, 10 mM HEPES, 10 mM glucose, and 4.93 mM Tris-base, pH 7.4 (95% O_2_, 5% CO_2_). The extracellular solution used for recording contained: 137 mM NaCl, 5.0 mM KCl, 1.0 mM CaCl_2_, 1.2 mM MgCl_2_, 10 mM HEPES, and 10 mM glucose, pH 7.4 (NaOH). The standard pipette-filling solution contained: 140 mM K-gluconate, 3.0 mM NaCl, 1.2 mM MgCl_2_, 10 mM HEPES, and 1.0 mM EGTA, pH 7.2 (KOH). Gramicidin (Sigma-Aldrich) was prepared from a stock solution (120 mg/1.0 ml DMSO) to a final concentration of 600 µg/ml of pipette-filling solution. Alternatively, amphotericin B was prepared from a stock solution (6 mg/100 µl DMSO) and added to a final concentration of 120 µg/ml of pipette-filling solution. Pipette tips were filled with a similar solution without gramicidin or amphotericin B. Cells were continuously (between test applications of agonists) perfused with gravity-fed extracellular solution provided by a custom-made pipette positioned 100-200 µm from the studied cell. Computer-controlled exchange to solutions containing agonist via solenoid valves occurred with a time constant of ∼50 ms, as measured by the change in offset potential on changing between high and low K^+^ concentrations in the perfusate (Karlsson et al., 2011).

### Electrophysiological recording

Electrophysiological experiments to study [Cl^–^]_i_ recovery were made by using the gramicidin-perforated patch technique, which enables recording of whole-cell currents without interference with the intracellular Cl^–^ concentration (Abe et al., 1994; Kyrozis and Reichling, 1995) and without current rundown (Wang et al., 2003). Experiments to estimate resting Cl^–^ conductance, which depend on estimates of [Cl^–^]_i_ but are compatible with slow Cl^–^ equilibration with the patch pipette, were made using the quicker amphotericin B-perforated patch technique (Rae et al., 1991). Both perforated-patch techniques are compatible with rapid Cl^–^ loading (Karlsson et al., 2011). The majority of experiments were made under voltage-clamp conditions, but a few control experiments were made under current-clamp conditions at zero current. Patch pipettes had a resistance of 3-4 MΩ when filled with standard pipette-filling solution and immersed in the standard extracellular solution (see above). Series resistance was evaluated repeatedly during experiments, by the “membrane test” provided in the Clampex software (versions 9 and 10; Molecular Devices), and was typically 15-40 MΩ. Online series-resistance compensation, which can never be complete and is associated with extra noise, was not used. For more complete compensation, all voltages were corrected for series resistance offline with voltage error computed from raw current and series resistance. Liquid‐junction potentials were calculated using the Clampex software and have been subtracted in all potentials given. All experiments were performed at room temperature (21-23°C).

### Loading cells with Cl^–^ and estimation of [Cl^–^]_i_ recovery time course

Cells were loaded with Cl^–^, to concentrations up to ∼70 mM, by using combined depolarization and application of GABA (1.0 mM) or glycine (1.0 mM; Karlsson et al., 2011). Estimates of [Cl^–^]_i_ during loading as well as during recovery after loading were calculated from *E*_Cl_ as obtained from *I*-*V* relations constructed from rapid voltage ramps (rate of ± 1.6 V s^−1^) during brief applications of GABA or glycine (100 µM to 1.0 mM) and after correction for series resistance and subtraction of leak currents. The studied cells generated stable (for >10 min) responses to GABA as well as to glycine during perforated-patch recordings (Druzin and Johansson, 2016). Since the method used to estimate [Cl^–^]_i_ depends on the current reversal potential, it is not sensitive to changes in the absolute GABA- or glycine-evoked conductance or peak current. Under the conditions used, without HCO_3_^–^ in the solutions, the reversal potentials for currents evoked by GABA or glycine (*E*_GABA_ and *E*_glycine_) are equivalent to *E*_Cl_ (Chavas and Marty, 2003). The probe applications of GABA/glycine were given at a voltage close to the estimated *E*_Cl_ to minimize influence on [Cl^–^]_i_. The charge transfer associated with a probe application of GABA/glycine was estimated to affect [Cl^–^]_i_ by <1.0 mM, as calculated from the “equivalent volume” of cytosol. The equivalent volume, v_equ_, was obtained from the Cl^–^ loading procedure and represented the volume in which the recorded charge transfer, Q, would cause the observed [Cl^–^]_i_ changes, Δ[Cl^–^]_i_, according to the relation: v_equ_ = QF^−1^ (Δ[Cl^–^]_i_)^−1^, where F is the Faraday constant. Thus, v_equ_ corresponds to the expected cytosolic volume (Karlsson et al., 2011). Leak currents, including small voltage-dependent components, were obtained from voltage-ramps given in the absence of GABA and glycine. The obtained leak *I*-*V* relation (after correction for series resistance) was fitted by a linear function or by the exponential function *I* = A + B *e^V^* ^/C^ (A, B, and C being constants). The latter function was used to calculate the leak current component when GABA or glycine was present. This procedure takes the differences in voltage, arising as a consequence of the series resistance and different current amplitudes, during ramps with and without GABA/glycine into account, and, therefore, provides a better estimate of leak currents than the commonly used technique to directly subtract the current recorded in the absence of ligand from that recorded in the presence of ligand. The similar results obtained with GABA and with glycine to load cells with Cl^–^ (Karlsson et al., 2011) show that the method used for loading is not critically influencing the results. Thus, GABA and glycine were here used interchangeably and each agonist was used for estimation of [Cl^–^]_i_ while blocking receptors for the other to clarify their role in [Cl^–^]_i_ regulation.

### Derivation of relative Cl^–^ conductance at rest

Previous methods for estimating the relative Cl^–^ conductance of the neuronal membrane at rest have not been straight-forward and dependent on many assumptions (Forsythe and Redman, 1988). We here aimed at a method with which we could estimate the relative Cl^–^ conductance from voltage ramps applied under conditions with different Cl^–^ concentrations on the in- or outside of the membrane. The starting assumption was that the membrane current reversal potential could be described by the “conductance” version of the Goldman-Hodgkin-Katz equation (Goldman, 1943; Hodgkin and Katz, 1949; see, e.g., Koester and Siegelbaum, 2013):

(1) $$E_{rev}=(g_{K}/g_{T})E_{K}+(g_{Na}/g_{T})E_{Na}+(g_{Cl}/g_{T})E_{Cl}$$

where *E*_rev_ is the reversal potential for the total current, i.e., the resting potential, *g* is conductance with subscripts T, K, Na, and Cl for total, K^+^, Na^+^, and Cl^–^, respectively, and *E*_K_, *E*_Na_, and *E*_Cl_ are the equilibrium potentials for respective ions. From Equation 1, we obtain

(2) $$g_{T}\;E_{rev}-g_{Cl}\;E_{Cl}=g_{K}\;E_{K}+g_{Na}\;E_{Na}$$

If we manipulate *E*_Cl_, e.g., by loading cells with Cl^–^ as described above, without changing ion conductances and without changing equilibrium potentials for Na^+^ and K^+^, then for the two conditions, before (subscript 1) and after (subscript 2) Cl^–^ loading,

(3) $$g_{T1}\;E_{rev1}-g_{Cl}\;E_{Cl1}=g_{T2}\;E_{rev2}-g_{Cl}\;E_{Cl2}$$

and if *g*_T_ does not change (as was experimentally verified, see below), then

(4) $$g_{T}\;E_{rev1}-g_{Cl}\;E_{Cl1}=g_{T}\;E_{rev2}-g_{Cl}\;E_{Cl2}$$

From Equation 4, we obtain the relative Cl^–^ conductance:

(5) $$g_{Cl}/g_{T}=(E_{rev2}-\;E_{rev1})/(E_{Cl2}-\;E_{Cl1})=\Delta E_{rev}/\Delta E_{Cl}$$

The relative Cl^–^ conductance may thus be obtained from the shift in reversal potential of total current relative to the shift in Cl^–^ equilibrium potential.

**Figure 1.**
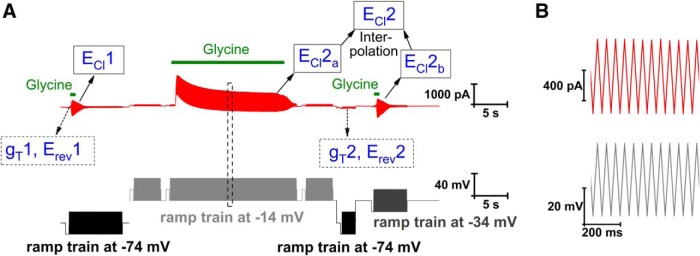
Protocol used for estimation of relative Cl^–^ conductance at rest. ***A***, Voltage protocol (below in black/gray; ramp sequences appearing as blocks; for detailed ramps, see ***B***) and recorded current (top, red), with glycine application as indicated by the green bars (short bars, 100 µM; long bar, 1.0 mM). ***B***, Voltage protocol (bottom) and recorded current (top) for part of the ramp train shown within dashed box in ***A***.

We here used continuous voltage ramp sequences, in a regular zigzag pattern, with application of glycine during part of the ramp sequence (Fig. 1). By this procedure, estimates of conductance and reversal potential were obtained before as well as throughout the glycine application, with a time resolution determined by the ramp cycle frequency, here 20 Hz. The control ramps (i.e., before or in the absence of glycine application) were used to estimate total conductance (*g*_T_) and reversal potential (*E*_rev_), but also for subtraction of leak currents from the currents evoked by glycine to estimate (*E*_Cl_). A first set of *g*_T_, *E*_rev_ and *E*_Cl_ estimates were obtained from voltage ramps symmetrically around a holding potential of –74 mV (i.e., near the resting potential). Subsequently the holding voltage was raised to –14 mV (with ramps symmetrically around this voltage, as indicated in Figure 1) and [Cl^–^]_i_ was raised by glycine (1.0 mM) application for 20 s. *E*_Cl_ was measured at the end of the 20-s glycine application (*E*_Cl_2_a_ in Figure 1). Next, holding voltage was returned to –74 mV and a second estimate of *g*_T_ (*g*_T_2) and *E*_rev_ (*E*_rev_2) was obtained. A final estimate of *E*_Cl_ (*E*_Cl_2_b_) was made with ramps around a holding voltage of –34 mV. A linear interpolation between *E*_Cl_2_a_ and *E*_Cl_2_b_ was used to estimate *E*_Cl_ at the time of *g*_T_2 (and *E*_rev_2), to account for [Cl^–^]_i_ changes during this time interval. *g*_T_2 was compared with *g*_T_1 to verify that glycine-activated conductance had decayed and to satisfy the conditions for Equation 4. Variations <30% between the two measurements in the same cell were accepted (to account for fluctuations in resting conductance), and absence of any significant systematic difference was confirmed (see Figure 5 below). These measurements were made with 100 µM Cd^2+^, to minimize transmitter release from adhering presynaptic terminals, and 30 mM TEA (replacing an equimolar amount of Na^+^ in the standard extracellular solution), to minimize voltage-gated K^+^ currents, tetrodotoxin (TTX; 2.0 µM), to block voltage-gated Na^+^ channels, and with the KCC2 blocker VU0255011-1 (10 µM) to minimize outward Cl^–^ transport between the times for measuring *E*_Cl_2_a_ and *E*_Cl_2_b_. To relate the derived resting *g*_Cl_ to the total membrane conductance in the absence of blockers, we also considered the effect of the used blockers on the total resting conductance *g*_T_. Control experiments showed that the total resting conductance in the presence of all four blockers (TEA, Cd^2+^, TTX, and VU0255011-1; *n =* 14) was, on average, only 54% (*p* = 0.0038, Mann–Whitney test) of that without blockers (*n =* 10). This effect could be ascribed to TEA and/or Cd^2+^ since the total conductance in a combination of TTX and VU0255011-1 (*n =* 14) was not significantly different from that without blockers (*n =* 10).

### Computation of [Cl^–^]_i_ recovery time course

The time course of theoretically expected [Cl^–^]_i_ recovery was computed for a simplified model of a spherical one-compartment cell (Karlsson et al., 2011). The model included Cl^–^ transport mediated by KCC2 as well as Cl^–^ current through a leak conductance. [Cl^–^]_i_ was computed numerically according to

(6) $$[Cl^{-}{]}_{i}\;(t+\Delta t)=[Cl^{-}{]}_{i}\;(t)+\Delta [Cl^{-}{]}_{i,\;KCC2}+\Delta [Cl^{-}{]}_{i,\;conductive}$$

where

(7) $$\Delta [Cl^{-}{]}_{i,\;KCC2}=-\Delta t\;\Delta \mu_{K,Cl}\;g_{KCC2}\;{v_{equ}}^{-1}\;(KCC2-mediated\;change\;in\;[Cl^{-}{]}_{i})$$

and

(8) $$\Delta [Cl^{-}{]}_{i,\;conductive}=I\Delta t\,{\text{ F}}^{-1}\;{v_{equ}}^{-1}\;(channel-mediated\;change\;in\;[Cl^{-}{]}_{i})$$

*t* is time, Δ*t* is the integration time step (here, 50 ms), *g*_KCC2_ is a transport proportionality factor (“apparent conductance”, dependent on the number and transport capacity of KCC2 molecules) for KCC2, Δ*µ*_K,Cl_ is the driving force for KCC2:

(9) $$\Delta \mu_{K,Cl}=RT\;(ln([Cl^{-}{]}_{i}/[Cl^{-}{]}_{o})+ln([K^{+}{]}_{i}/[K^{+}{]}_{o})),$$

and

(10) $$I=g_{Cl}\;(V_{m}-E_{Cl})\;\;\;\;\;\;\;\;({Cl}^{-}\;leak\;current),$$

**Figure 2.**
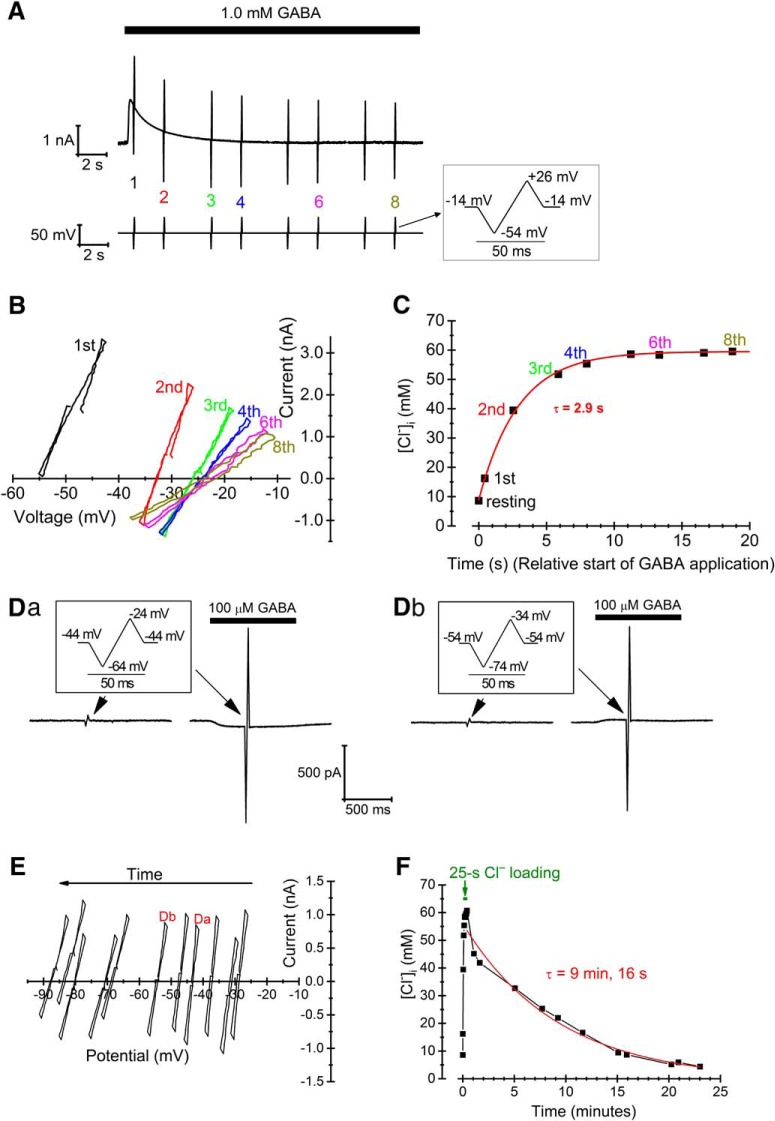
[Cl^–^]_i_ loading and estimation of transporter-dependent recovery capacity. ***A***, Voltage protocol (bottom) and recorded current (top) during loading a cell with Cl^–^. Note that the baseline voltage during loading was –14 mV to obtain a large driving force for Cl^–^ current evoked by 1.0 mM GABA. Data from numbered ramps are shown in ***B*** and ***C***. ***B***, *I-V* relations corresponding to the ramps (no 1-4, 6, 8, as marked in ***A***) applied during Cl^–^ loading (corrected for series resistance and with leak components subtracted). ***C***, Time course of [Cl^–^]_i_ during Cl^–^ loading, as computed from *E*_Cl_ obtained from the *I-V* relations in ***B***, and superimposed fitted exponential curve. Resting [Cl^–^]_i_ was obtained from a separate, preceding ramp (not shown). ***D***, Voltage ramps (insets), leak and GABA-evoked currents used to probe [Cl^–^]_i_ during recovery after the end of Cl^–^ loading. Ramps for [Cl^–^]_i_ estimates at two different times are shown in ***Da*** and ***Db*** (compare *I*-*V* relations in ***E***). ***E***, *I-V* relations from probe currents as in ***D*** (with the relations corresponding to currents in ***Da*** and ***Db*** indicated). Note that *I-V* relations are shifted with time to more negative voltages, as a consequence of the changing [Cl^–^]_i_ and *E*_Cl_. ***F***, Full-time course of [Cl^–^]_i_ changes during loading (first 25 s) and recovery phases with superimposed mono-exponential fit (red thick line) of the recovery phase.

F is the Faraday constant and v_equ_ is the equivalent volume, i.e., the cytosolic volume where Cl^−^ equilibrates, taken to be 50% of the total cell volume (here, assumed 5.0 10^−13^ l, i.e., 50% of the volume of a sphere of radius 6.2 µm; cf Karlsson et al., 2011). The proportionality factor *g*_KCC2_ was adjusted to match KCC2-mediated [Cl^–^]_i_ recovery to experimental results (experiments with minimized influence of *g*_Cl_, compare Fig. 2*F*), whereas *g*_Cl_ was as experimentally recorded (compare Figure 5 below). The computations were made using Turbo Basic software (Borland).

### Statistical analysis

Data are presented as the mean ± SEM, with *n* representing the number of cells studied. Differences between groups were analyzed with the paired Wilcoxon signed-rank test for paired data and with the Mann–Whitney test for unpaired data; *p* < 0.05 was considered statistically significant. Statistical analyses were performed using the software OriginPro 2016 (OriginLab).

## Results

### Transporter-dependent recovery of [Cl^–^]_i_

To obtain an overall view of the [Cl^–^]_i_ recovery capacity in dissociated medial preoptic nucleus (MPN) neurons, we first addressed transporter-dependent recovery after a Cl^–^ load to concentrations significantly above baseline [Cl^–^]_i_, previously estimated to ∼9 mM in this preparation (Karlsson et al., 2011). To estimate the capacity, we loaded the cells with Cl^–^ by combined depolarization and GABA or glycine application. This resulted in an exponential (time constant 3.8 ± 0.4 s, *n =* 19) increase in [Cl^–^]_i_ to 60 ± 3 mM (*n =* 19; Fig. 2*A-C*). After loading, [Cl^–^]_i_ recovery was studied by voltage ramps (to estimate *E*_Cl_) given from a holding potential that was kept as close as possible to the gradually changing *E*_Cl_, to minimize influence of passive conductive recovery and also to minimize influence of the GABA/glycine applications during recovery (Fig. 2*D*,*E*). This required test runs to obtain a rough estimate of the *E*_Cl_ recovery time course. Under these conditions, favoring mainly nonconductive, transporter-dependent changes, [Cl^–^]_i_ recovery varied somewhat between cells, following a roughly exponential, a doubly exponential or a nearly linear time course (Fig. 2*F*). For simplicity, we used the best fitted single-exponential for comparison between cells and recording conditions. In control conditions, the time constant was 499 ± 49 s (*n =* 19). Since the recovery time course was considerably slower than estimated for some other cell types, and with other techniques (Staley and Proctor, 1999; Wagner et al., 2001; Földy et al., 2010; Rinke et al., 2010; Deisz et al., 2011), we made a few recordings from neurons dissociated from the CA1 region in brain slices containing the hippocampus. However, in the three hippocampal cells tested, [Cl^–^]_i_ recovery occurred with a time constant of 1877 ± 60 s, thus even slower than for the MPN neurons.

**Figure 3.**
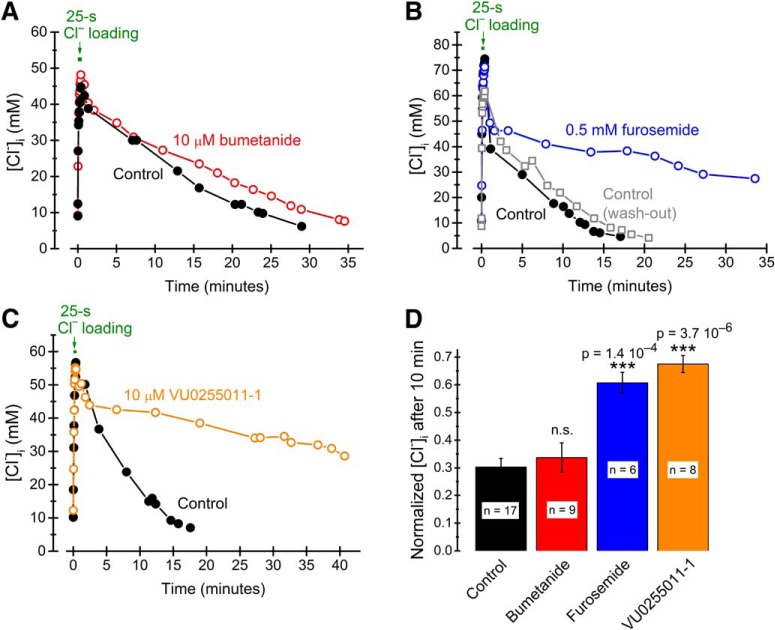
Pharmacology of transporter-mediated recovery of [Cl^–^]_i_. ***A***–***C***, [Cl^–^]_i_ recovery under control conditions (black filled circles) and in the presence of 10 µM bumetanide (***A***, red open circles), 0.5 mM furosemide (***B***, blue open circles), and 10 µM VU0255011-1 (***C***, orange open circles), with control and drug application for the same cell. Cl^–^ was loaded and [Cl^–^]_i_ quantified as in Figure 2. ***D***, Summary of drug effects on the level of [Cl^–^]_i_ after 10 min recovery, measured as [Cl^–^]_i_ normalized to maximal [Cl^–^]_i_ during Cl^–^ loading. Note the lack of significant effect of the NKCC1-blocker bumetanide but highly significant effects of the KCC2 blockers furosemide and VU0255011-1.

To verify that the [Cl^–^]_i_ recovery described was mediated by transporter activity, we applied several types of blockers. The time course of recovery was not significantly affected by a blocker (bumetanide, 10 µM) of the Na^+^-K^+^-Cl^–^ cotransporter 1 (NKCC1; Fig. 3*A*,*D*) but was dramatically slowed by furosemide (0.5 mM; Fig. 3*B*,*D*), which affects also KCC2, as well as by the selective KCC2 blocker VU0255011-1 (10 µM; Fig. 3*C*,*D*). These effects are consistent with the idea that a major fraction of the observed recovery is mediated by KCC2. The kinetic details of transporter-dependent [Cl^–^]_i_ recovery were not investigated further here.

### Passive, conductive recovery of [Cl^–^]_i_

**Figure 4.**
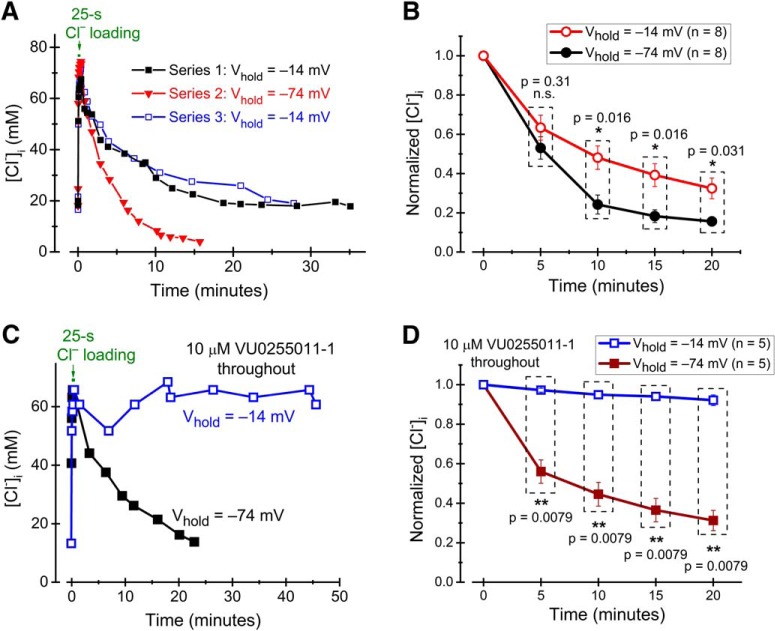
Effect of voltage on [Cl^–^]_i_ recovery. ***A***, [Cl^–^]_i_ recovery in an individual cell at two different levels of holding voltage, as indicated, in control solution. Note that [Cl^–^]_i_ recovers quicker and to a lower concentration at –74 mV compared with at –14 mV. ***B***, Summary of data showing [Cl^–^]_i_ recovery at –74 and at –14 mV (as in ***A***) in eight neurons. ***C***, [Cl^–^]_i_ recovery in an individual cell at two different levels of holding voltage, as indicated, in the presence of 10 µM VU0255011-1. ***D***, Summary of data showing [Cl^–^]_i_ recovery at –74 and at –14 mV in the presence of 10 µM VU0255011-1 (as in ***C***) in five neurons. Probe pulses to estimate [Cl^–^]_i_ were given at slightly different times during each recovery interval (to keep the membrane potential close to *E*_Cl_). To compare at similar times, in ***B*** and ***D***, interpolation was used for data pairs closest to the time points illustrated, and [Cl^–^]_i_ was normalized to the maximal concentration during loading.

If a substantial conductive component contributes to [Cl^–^]_i_ recovery, this should be seen as a voltage-dependent component. KCC2-mediated Cl^–^ transport does not depend on voltage (see, e.g., Alvarez-Leefmans and Delpire, 2009). We therefore studied [Cl^–^]_i_ recovery as above, but with membrane voltage kept at either –14 or –74 mV during the recovery phase. Test pulses of GABA/glycine were still applied at a holding voltage close to *E*_Cl_, to minimize GABA/glycine-induced Cl^–^ flux. The time at these voltages was, however, very small compared with the total recovery time. The recovery time constant observed at –14 mV was 843 ± 208 s (*n =* 8), significantly (*p* = 0.0078; paired Wilcoxon signed-rank test) longer than that at –74 mV (375 ± 95 s; *n =* 8) in the same cells. The difference obtained by changing the holding voltage was reversible (Fig. 4*A*,*B*). In addition, [Cl^–^]_i_ recovered to a lower level (4.3 ± 1.2 mM, *n =* 8) at –74 mV than at –14 mV (10.9 ± 3.0 mM; *n =* 8; *p* = 0.039; evaluated from the asymptote of the fitted exponential function). Thus, the data are compatible with a substantial contribution of conductive Cl^–^ flux to [Cl^–^]_i_ recovery.

To quantify the conductive component of [Cl^–^]_i_ recovery in isolation, we repeated the experiments with different holding voltages in the presence of the KCC2 blocker VU0255011-1 (10 µM): There was only little recovery (7.9 ± 2.7% of the loaded [Cl^–^]_i_ recovered after 20 min; *n =* 5) at –14 mV, whereas at –74 mV, [Cl^–^]_i_ recovered with a time constant of 651 ± 171 s (*n =* 5) and 60 ± 5% of the [Cl^–^]_i_ increase during loading was eliminated after 20 min of recovery (Fig. 4*C*,*D*). Thus, it is clear that without KCC2 there is very small, if any, Cl^–^ extrusion capacity to counteract the effect of conductive Cl^–^ transport on [Cl^–^]_i_ at –14 mV and that at –74 mV, conductive Cl^–^ transport accounts for a recovery time course in the same order of magnitude as does KCC2 in the absence of blockers (compare Fig. 3, black filled symbols, with Fig. 4*D*, brown filled symbols).

### Estimate of resting Cl^–^ conductance

**Figure 5.**
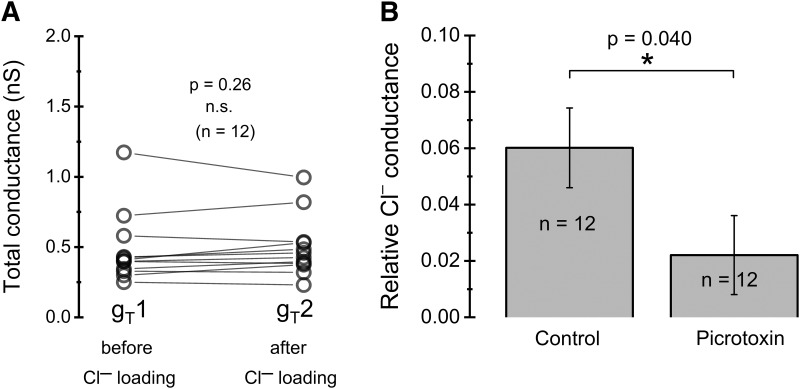
Relative resting Cl^–^ conductance. ***A***, Distribution of measured total conductances (in the presence of VU0255011-1, TTX, Cd^2+^, and TEA; see main text), showing no significant difference before and after Cl^–^ loading, as required for the method used to estimate resting Cl^–^ conductance. Data paired for individual cells. ***B***, Relative resting Cl^–^ conductance, in control conditions and in the presence of 100 µM PTX. Note that the given values in ***B*** are relative to the total membrane conductance in the absence of blockers.

From above, it is clear that although KCC2 is required for a rapid and complete recovery of [Cl^–^]_i_ to normal resting levels, a significant recovery takes place also by passive, conductive pathways in the absence of functional KCC2. The rate of such recovery will depend on the conductance to Cl^–^, *g*_Cl_. Resting *g*_Cl_ will also be an important determinant of resting [Cl^–^]_i_ (Johansson et al., 2016). We therefore derived a method for estimating the resting *g*_Cl_, as described in Materials and Methods and Figure 1. Since the method depends on a total membrane conductance, *g*_T_, that does not change with Cl^–^ loading, we first verified that *g*_T_ before (*g*_T_1) and shortly after (*g*_T_2) Cl^–^ loading was not significantly different (Fig. 5*A*). Subsequently, we computed the relative resting *g*_Cl_, from Equation 4 and the conductance and reversal potential measures obtained as described in Figure 1. A final computation accounted for the effect of used background blockers (TEA, Cd^2+^, TTX, and VU0255011-1) on the total membrane conductance (see Materials and Methods). The obtained relative resting *g*_Cl_ was 6.0 ± 1.4% (*n =* 12; relative to total resting conductance in the absence of blockers), corresponding to an absolute resting *g*_Cl_ of 54 ± 13 pS (*n =* 12).

We next addressed the molecular basis for the resting *g*_Cl_. We first tested the hypothesis that open GABA_A_ receptors accounted for part of *g*_Cl_. Relative resting *g*_Cl_ obtained from experiments with PTX (100 µM) added to the external solution was only 2.2 ± 1.4% (*n =* 12; Fig. 5*B*), or ∼37% of that without PTX. Since 200 µM PTX does not significantly affect the near-steady glycine-evoked current in the studied cell type (Karlsson et al., 2011), this suggests that GABA_A_ receptors account for a major fraction of resting *g*_Cl_.

### ClC-2 channels do not contribute significantly to resting conductance in MPN neurons

**Figure 6.**
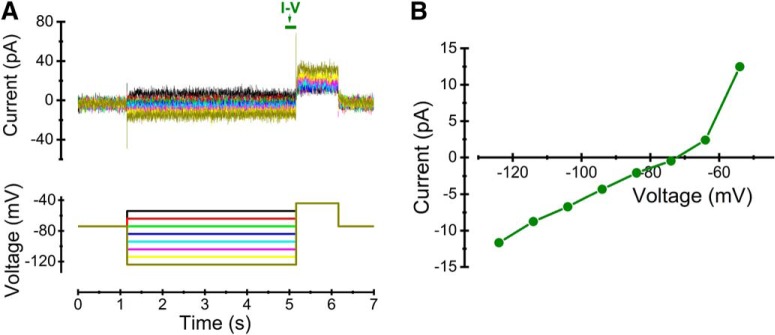
Lack of ClC-2 currents. ***A***, Voltage protocol (bottom) used for detecting ClC-2 currents and corresponding currents (top) recorded in an MPN neuron. Colors of individual current traces matches the colors in the voltage protocol. ***B***, *I*-*V* relation for the currents in ***A*** (average amplitude during the time marked by olive bar in ***A***). Note the small amplitude and the linearity in the range below –65 mV.

Because Cl^–^-permeable ClC-2 channels have been reported to contribute substantially to the resting conductance in CA1 pyramidal cells (Rinke et al., 2010), we used a voltage protocol suitable for detection of Cl^–^ currents through ClC-2 channels (Fig. 6*A*, bottom) to establish whether the MPN neurons express such channels. However, the current responses in eight cells tested were only composed of linear capacitive and small leak currents in the relevant voltage range (Fig. 6*A*, top, *B*), making it unlikely that ClC-2 contributes significantly to the resting conductance in MPN neurons.

### Contribution of GABA_A_ receptors to conductive [Cl^–^]_i_ recovery

**Figure 7.**
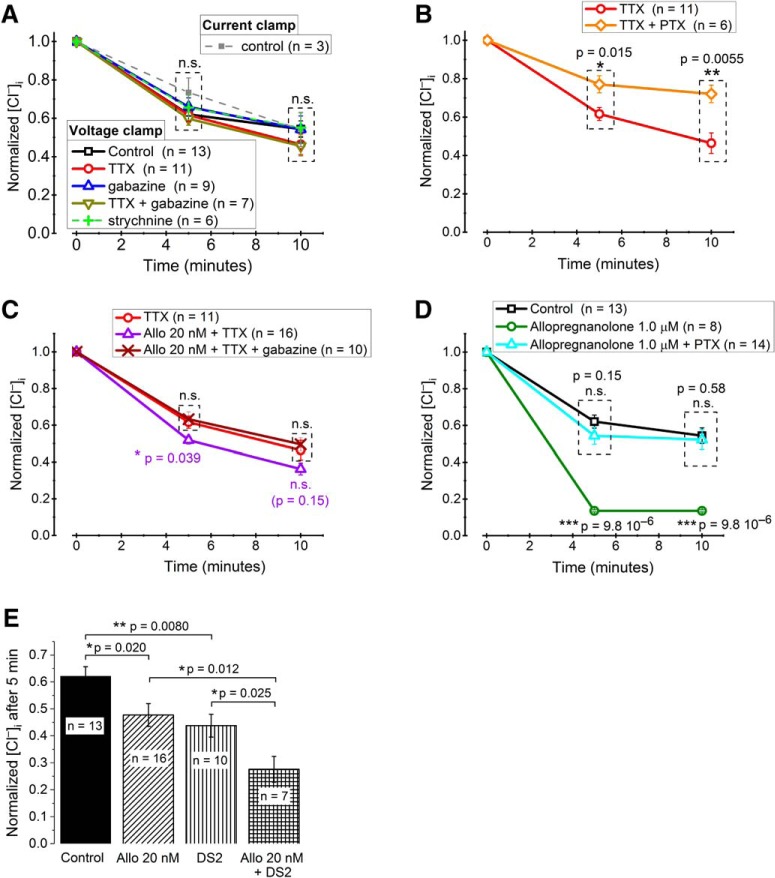
Role of spontaneously open GABA_A_ receptors and of neurosteroid-potentiated mIPSCs for [Cl^–^]_i_ recovery. ***A***, Relative [Cl^–^]_i_, illustrating recovery 5 and 10 min after Cl^–^ loading. Current-clamp (0 pA) between test ramps during recovery phase: control only. Voltage-clamp (–74 mV) between test ramps during recovery phase: control, 2.0 µM TTX, 3.0 µM gabazine, a combination of TTX and gabazine, or 10 µM strychnine, as indicated. Note the lack of significant differences. ***B***, Relative [Cl^–^]_i_ (as in ***A***, voltage-clamp only), with significantly reduced recovery in the presence of 100 µM PTX. ***C***, Relative [Cl^–^]_i_ (as in ***B***), showing gabazine-sensitive enhancement of recovery in the presence of 2.0 µM TTX and the neurosteroid allopregnanolone (Allo) in a concentration (20 nM) known to enhance mIPSC frequency and prolong mIPSC decay. ***D***, Relative [Cl^–^]_i_ (as in ***B***) showing the dramatic enhancement of [Cl^–^]_i_ recovery by allopregnanolone in a concentration (1.0 µM) that may directly activate GABA_A_ receptors and the block of this effect by 100 µM PTX. ***E***, Comparison of the effects of 20 nM Allo, DS2 and a combination of Allo and DS2 on normalized [Cl^–^]_i_ after 5 min of recovery from a Cl^–^ load as in ***A***–***D***. Note the highly significant effect of DS2 and the additive effects of Allo and DS2. To compare data recorded at slightly different times in ***A***–***E***, interpolation was used for data pairs closest to the time points illustrated and [Cl^–^]_i_ was normalized to the maximal concentration during loading.

The above results show that there is a significant resting Cl^–^ conductance in MPN neurons and suggest that a major fraction of this conductance is due to GABA_A_ receptors. We therefore expected that GABA_A_ receptors may contribute to [Cl^–^]_i_ recovery after a high Cl^–^ load. In dissociated MPN neurons, GABA_A_ receptors may be activated as a consequence of spontaneous GABA release from adhering presynaptic nerve terminals (Haage et al., 1998). We speculated that possibly such spontaneous GABA release could contribute to *g*_Cl_ and [Cl^–^]_i_ recovery. To clarify this, we studied the time course of conductive [Cl^–^]_i_ recovery (i.e., in the presence of 10 µM VU0255011-1 to block KCC2) under voltage-clamp conditions at –74 mV. A few control recordings showed that in the absence of additional blockers, a roughly similar time course of [Cl^–^]_i_ recovery is seen under current-clamp conditions at zero current (Fig. 7*A*). We first compared [Cl^–^]_i_ recovery at –74 mV without and with TTX (2.0 µM) to block action potential-evoked release of GABA, however, without noting any significant difference (Fig. 7*A*). We subsequently added 3.0 µM gabazine (SR-95531 hydrobromide), previously shown to block spontaneous IPSCs in the studied cell type (Karlsson et al., 2011), but also this was without significant effect on conductive [Cl^–^]_i_ recovery, as was also the glycine-receptor blocker strychnine (10 µM; Fig. 7*A*). The lack of effect of gabazine was somewhat surprising in light of the above suggested contribution of GABA_A_ receptors to resting *g*_Cl_. However, while PTX, which was used above for resting *g*_Cl_, is noncompetitive and usually considered as a pore blocker (Takeuchi and Takeuchi, 1969; Etter et al., 1999), gabazine is a competitive GABA_A_ receptor antagonist (Heaulme et al., 1986; Hamann et al., 1988) and may fail to block receptor-channels that are spontaneously open in the absence of GABA (Birnir et al., 2000; Wlodarczyk et al., 2013). Indeed, consistent with the effect of PTX on resting *g*_Cl_, PTX significantly reduced conductive [Cl^–^]_i_ recovery in the studied 10-min interval (Fig. 7*B*), suggesting that spontaneously open GABA_A_ receptors in the absence of GABA do contribute to recovery.

### Neurosteroid regulates conductive [Cl^–^]_i_ recovery via miniature IPSCs (mIPSCs)

Although the above results suggested that spontaneous, action potential-independent GABA release does not significantly affect [Cl^–^]_i_ recovery, we hypothesized that such release and the generated mIPSCs may still play a physiologic role for the Cl^–^ homeostasis. Hypothetically, mIPSC potentiation by an endogenous modulator may enable such a functional role for the spontaneously released transmitter. The endogenous neurosteroid 3α-hydroxy-5α-pregnane-20-one (allopregnanolone), which may be produced *de novo* in the brain, e.g., during stress (Purdy et al., 1991), increases the frequency as well as prolongs the decay time of mIPSCs in MPN neurons (Haage and Johansson, 1999). We therefore analyzed the effect of allopregnanolone on [Cl^–^]_i_ recovery at –74 mV in the presence of 10 µM VU0255011-1 to block KCC2. It was clear that, in the presence of TTX (2.0 µM) to block action potential-evoked GABA release, 20 nM allopregnanolone, a concentration that mainly affects the time course of mIPSCs (Haage et al., 2002) and may be reached *in vivo* (Purdy et al., 1991), significantly potentiated [Cl^–^]_i_ recovery as quantified 5 min after loading with Cl^–^ (Fig. 7*C*). This effect of allopregnanolone was abolished by gabazine (3.0 µM; Fig. 7*C*), suggesting that the steroid effect was mediated by GABA_A_ receptors that were activated by GABA, as expected for mIPSCs. Allopregnanolone at 20 nM concentration did not affect the baseline current in 11 cells tested (*p* = 0.55; paired sample Wilcoxon signed-rank test), ruling out significant effects on tonic GABA_A_ receptor-mediated currents at this steroid concentration.

Higher, micromolar concentrations of allopregnanolone may directly activate GABA_A_ receptors in MPN neurons (Haage and Johansson, 1999). Here, the effect of 1.0 µM allopregnanolone on [Cl^–^]_i_ recovery was dramatic and highly significant (Fig. 7*D*, compare top and lower curves). The effect was abolished in the presence of 100 µM PTX, confirming an action on GABA_A_ receptors (Fig. 7*D*).

It seems possible that some of the effect of 1.0 µM allopregnanolone may be mediated by δ-subunit-containing GABA_A_ receptors, which are extra- or peri-synaptically located and highly sensitive to potentiation by neuroactive steroids (Stell et al., 2003). They may also display a degree of spontaneous activity (Jensen et al., 2013; Wlodarczyk et al., 2013) that should contribute to the background Cl^–^ conductance. We took advantage of the positive allosteric modulator δ-selective compound 2 (DS2; Wafford et al., 2009; Jensen et al., 2013), which, although not perfectively selective, may preferentially potentiate δ-subunit-containing GABA_A_ receptors at concentrations <2 µM (Ahring et al., 2016). Here, DS2 (1.0 µM) significantly enhanced the [Cl^–^]_i_ recovery as measured 5 min after loading with Cl^–^ (Fig. 7*E*), suggesting that [Cl^–^]_i_ recovery may be pharmacologically enhanced via positive modulation of δ-subunit-containing GABA_A_ receptors. This effect was additive to the effect of 20 nM allopregnanolone (Fig. 7*E*).

### Theoretically expected influence of conductive Cl^–^ flux on [Cl^–^]_i_ recovery

**Figure 8.**
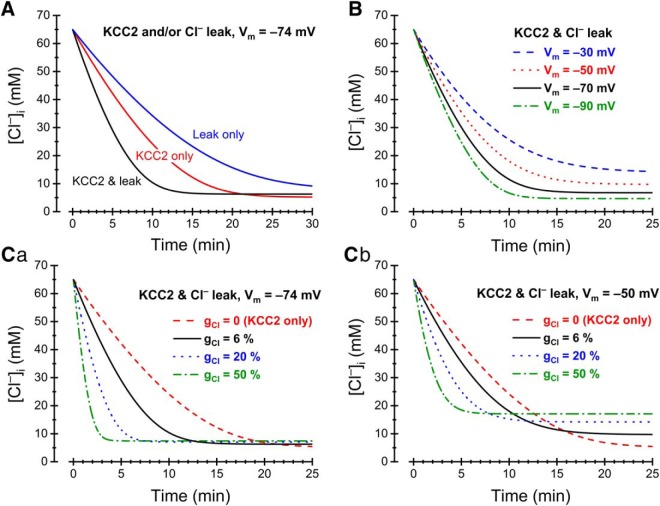
Computed [Cl^–^]_i_ recovery. Dependence on *g*_Cl_, *g*_KCC2_, and *V*_m_. ***A***, Recovery mediated by KCC2 only (red, middle), Cl^–^ leak only (blue, top), or a combination of KCC2 and Cl^–^ leak (black, bottom) at *V*_m_ = –74 mV. ***B***, Recovery mediated by KCC2 and Cl^–^ leak in combination at different *V*_m_, as indicated. Note that the rate of recovery as well as the asymptotic steady-state [Cl^–^]_i_ depends on *V*_m_. ***C***, Recovery mediated by KCC2 and Cl^–^ leak in combination at different relative *g*_Cl_, as indicated and at *V*_m_ = –74 mV (***Ca***) or *V*_m_ = –50 mV (***Cb***). Note that the speed of recovery increases with *g*_Cl_ and that the asymptotic steady-state [Cl^–^]_i_ also increases with *g*_Cl_. The latter effect is weak at –74 mV (***Ca***) but prominent at –50 mV (***Cb***). The transport capacity of KCC2 in (***A–C***) was adjusted by setting the transporter proportionality factor (apparent conductance; Johansson et al., 2016) *g*_KCC2_ to 6.7 10^−21^ mol^2^ V^−1^ C^−1^ s^−1^, to match modeled KCC2-mediated [Cl^–^]_i_ recovery time course to experimental results.

The experimental results described above show that MPN neurons display a resting Cl^–^ conductance that contributes significantly to recovery of [Cl^–^]_i_ after a high Cl^–^ load. To clarify the theoretically expected contribution of the resting *g*_Cl_ to [Cl^–^]_i_ recovery, we used the model described by Karlsson et al. (2011) to compute the time course of [Cl^–^]_i_. In essence, the influence of relative *g*_Cl_ and of the KCC2-mediated transport capacity (*g*_KCC2_) was analyzed in a single-compartment cell with dimensions, total resting conductance and ion (external K^+^ and Cl^–^, internal K^+^) concentrations matching experimental values (see Materials and Methods). The time course of computed [Cl^–^]_i_ recovery mediated by KCC2 in the absence of resting *g*_Cl_ was matched to the experimental results (Fig. 2*F*, control; experiment with minimized influence of *g*_Cl_) by adjusting *g*_KCC2_ (Fig. 8*A*, red, middle).

Assuming a relative resting *g*_Cl_ of 6.0% (of a total conductance of 0.89 nS in blocker-free conditions, to match the experimentally obtained conductance of 0.48 nS in Figure 5*A*, left, and the effect of the used blockers on *g*_T_ as described in Materials and Methods), but no KCC2-mediated transport resulted in a computed [Cl^–^]_i_ recovery (at –74 mV) that reasonably well matched the experimental recovery (Fig. 4*D*, lower curve) observed with KCC2 blocked (Fig. 8*A*, blue, top). The computed [Cl^–^]_i_ recovery with *g*_Cl_ as well as KCC2-mediated transport (Fig. 8*A*, black, bottom) was quicker, showing that even with a relative resting *g*_Cl_ as low as 6.0%, a substantial effect on [Cl^–^]_i_ is indeed expected. It is also clear that [Cl^–^]_i_ settles at a slightly higher steady level due to the combined effects of *g*_Cl_ and KCC2 as compared with KCC2-mediated transport only (Fig. 8*A*, black, bottom). It was recently shown that, in a cell where [Cl^–^]_i_ is determined by *g*_Cl_ and *g*_KCC2_, at steady-state, *E*_Cl_ will settle between the membrane potential and the equilibrium potential for K^+^, depending on the relative influence of *g*_Cl_ and of *g*_KCC2_ (Johansson et al., 2016). As shown here (Fig. 8*B*), the membrane potential (*V*_m_) influences the rate of [Cl^–^]_i_ recovery as well as the steady-state [Cl^–^]_i_. As *g*_Cl_ drives *E*_Cl_ toward *V*_m_, there is a trade-off between the rate of [Cl^–^]_i_ recovery (maximal at high *g*_Cl_) and the steady-state [Cl^–^]_i_ (minimal at low *g*_Cl_). The effect of *g*_Cl_ on steady-state [Cl^–^]_i_ is small at strongly negative *V*_m_ (–74 mV; Fig. 8*Ca*) but prominent at less negative *V*_m_ (–50 mV; Fig. 8*Cb*). It is clear from Figure 8*C* that, at more negative potentials in particular, there is a strong potential to modulate the rate of [Cl^–^]_i_ recovery by substances that affect *g*_Cl_, such as neuroactive steroids or GABA_A_ receptor active compounds like DS2 and PTX. At more positive potentials, in particular, there is also a potential to modulate resting [Cl^–^]_i_ by such substances.

## Discussion

In the present study, we have used voltage-ramp techniques to analyze how passive, conductive Cl^–^ flux contributes to [Cl^–^]_i_ recovery after a significant Cl^–^ load introduced in central neurons. We have also compared with [Cl^–^]_i_ recovery mediated by the transporter KCC2 and showed that conductive flux may account for a contribution in the same order of magnitude as KCC2. To estimate the relative resting Cl^–^ conductance, for which little information is available concerning central mammalian neurons, we developed a novel technique based on [Cl^–^]_i_ perturbation. We showed a relative resting Cl^–^ conductance of 6%, with a major fraction mediated by open GABA_A_ receptors. We also showed that conductive [Cl^–^]_i_ recovery may be dramatically potentiated by the endogenous neurosteroid allopregnanolone, acting by enhancing the frequency and prolonging the time course of miniature postsynaptic currents. This suggests a novel role for neurosteroids in Cl^–^ homeostasis as well as a novel role for spontaneous transmitter release.

### KCC2-mediated [Cl^–^]_i_ recovery in isolated preoptic neurons

The time constant of [Cl^–^]_i_ recovery in the studied MPN neurons was considerably longer than the few seconds reported in some earlier studies (Staley and Proctor, 1999; Wagner et al., 2001; Földy et al., 2010; Rinke et al., 2010; Deisz et al., 2011), including studies of hippocampal CA1 neurons, where whole-cell recording or sharp microelectrodes were used. The recordings we made from hippocampal neurons showed a still slower [Cl^–^]_i_ recovery, suggesting that the difference from the earlier studies did not depend on the cell type, but likely on the recording techniques or type of preparational procedures. It seems likely that earlier studies with sharp microelectrodes or whole-cell recording may not have fully accounted for the introduced Cl^–^ leak to/from the outside or equilibration with the patch pipette, the latter which may occur within a few seconds (Pusch and Neher, 1988). Further, the negative holding voltage used in some studies implies that conductive [Cl^–^]_i_ recovery may have contributed significantly, whereas we reduced influence of conductive recovery by changing the holding voltage gradually while transporter-mediated recovery was estimated. Indeed, studies using the gramicidin-perforated patch technique report a slower recovery (>30 s up to several minutes; Chabwine et al., 2004; Lee et al., 2011; Pellegrino et al., 2011). Also measurements with optical Cl^–^ sensors suggest a recovery time constant of ∼30 s (Berglund et al., 2006) or several minutes (Friedel et al., 2015), although conductive recovery may have contributed also there. On the other hand, possibly the KCC2-mediated transport is dependent on the naturally surrounding milieu, which was largely eliminated by the dissociation process in the present work. Clearly, dendrites were removed in the present study. The [Cl^–^]_i_ fluctuations in dendrites are expected to be much quicker than in the cell body (Doyon et al., 2011). Therefore, the presently reported time course of [Cl^–^]_i_ recovery should be interpreted as reflecting the KCC2-dependent transport capacity of isolated neuronal cell bodies.

### Conductive [Cl^–^]_i_ recovery

For any cell with a significant Cl^–^ permeability, a raised cellular [Cl^–^]_i_ is expected to recover by conductive pathways to a level determined in part by the membrane potential. To our knowledge, such recovery has previously not been rigorously quantified. We here showed that, surprisingly, conductive [Cl^–^]_i_ recovery at negative holding potentials was substantial, with a time course roughly similar to the time course of isolated KCC2-dependent recovery. This implies that the gain in speed of recovery contributed by KCC2 is not dramatic, if a sufficiently negative resting potential can be maintained. Nevertheless, KCC2 has been shown important for nervous function, with reduced KCC2 function being associated with several types of pathology, such as neuropathic pain (Coull et al., 2003), epilepsy (Huberfeld et al., 2007), autism (Tyzio et al., 2014; Merner et al., 2015), schizophrenia (Merner et al., 2015), spasticity (Boulenguez et al., 2010), and ischemia (Jaenisch et al., 2010). There are several possibilities as to why KCC2 may be needed despite substantial conductive recovery. First, conductive recovery is dependent on the membrane potential, *V*_m_. Thus, when *V*_m_ is raised, e.g., as a consequence of ongoing excitatory synaptic activity, conductive Cl^–^ flux will drive [Cl^–^]_i_ toward a higher level, where *E*_Cl_ equals the momentary *V*_m_. In this respect, conductive Cl^–^ flux may contribute to loading of Cl^–^ at raised *V*_m_ (as it does during the loading procedure used in the present work). Thus, KCC2 may be crucial in conditions of raised *V*_m_. Second, even at the resting potential in the absence of synaptic depolarization, conductive [Cl^–^]_i_ recovery cannot lower *E*_Cl_ beyond the membrane potential, and thus not sufficiently to produce hyperpolarizing GABA or glycine responses. The slight HCO_3_^–^ permeability of GABA_A_ and glycine receptors implies that extra lowering of *E*_Cl_ is needed for hyperpolarizing responses. Although GABA and glycine could still mediate shunting inhibition, it seems possible that hyperpolarizing responses may be of advantage in some conditions, due, e.g., to the different distribution in time and space of hyperpolarization and the underlying conductance change (Kaila et al., 2014). Third, the contribution by KCC2 to [Cl^–^]_i_ recovery, even if small, still may be crucial to normal neural function. The finding that even mild KCC2 hypofunction may degrade neural coding (Doyon et al., 2016a) supports this possibility. Since conductive [Cl^–^]_i_ recovery may be of a similar magnitude as KCC2-dependent recovery, it seems likely that it will also be critical for neural coding and that reduced conductive recovery may be associated with pathology. Further, the potential for modulation of [Cl^–^]_i_ recovery via altered *g*_Cl_, as illustrated in Figure 8*Ca*, suggests that the levels of neuroactive steroids or the presence of other GABA_A_ receptor active substances, such as DS2, may be expected to affect the degree of disturbance in [Cl^–^]_i_-related pathologic conditions.

The expression of Cl^–^ transporters and [Cl^–^]_i_ change during development, with fetal and early postnatal stages dominated by a low expression of KCC2, but a relatively high expression of NKCC1 and a relatively high [Cl^–^]_i_ (see, e.g., Ben-Ari, 2002). If [Cl^–^]_i_ is to be maintained lower than dictated by the thermodynamic equilibrium for NKCC1 (usually >60 mM), NKCC1 must be opposed by outward Cl^–^ flux. In the absence of Cl^–^ extruding transporters, the resting *g*_Cl_ in combination with a negative resting potential may account for such outward Cl^–^ flux. Thus, we may expect that conductive [Cl^–^]_i_ recovery plays a relatively larger role early in development and in other situations when KCC2 expression is low, compared with when KCC2 expression is high.

### Resting Cl^–^ conductance

The rate of conductive [Cl^–^]_i_ change will necessarily depend on the membrane Cl^–^ conductance, *g*_Cl_. Neuronal membranes are permeable to Cl^–^ even at rest (Russell and Brown, 1972; Strickholm and Clark, 1977), but for mammalian neurons, information on the quantity of the resting Cl^–^ permeability or *g*_Cl_ is scarce. Such information is needed not only to understand recovery of a perturbed [Cl^–^]_i_. The background *g*_Cl_ is also critically influencing neuronal firing properties, such as action potential frequency adaptation and “after discharge”, and the control of cell volume (Berndt et al., 2011). Forsythe and Redman (1988) calculated the approximate resting *g*_Cl_ in spinal motorneurons to 18% of the total (Na^+^, K^+^, and Cl^–^) conductances, but their estimate depended on several assumptions, including unknown changes in K^+^ conductance, and they emphasize the obstacles to estimation introduced by the complex neuronal morphology. In the present work, the latter type of obstacles was avoided by using isolated neuronal cell bodies. Further, the novel method we developed for quantifying *g*_Cl_ employed a simple expression (Eq. 5), which depended mainly on the testable and verified assumption of constant total conductance in the experimental conditions used. The obtained resting *g*_Cl_ was relatively low (mean relative *g*_Cl_ 6% and absolute *g*_Cl_ 54 pS), with a major (∼2/3) contribution by GABA_A_ receptors, as suggested by the reducing effect of PTX. This corresponds to the conductance of only two open GABA_A_ receptor channels (with most frequent single-channel conductance of 17 pS; Bormann et al., 1987). Since the presence of δ-subunit-containing GABA_A_ receptors, which may contribute to tonic currents (Jensen et al., 2013; Wlodarczyk et al., 2013), was suggested above (see Results), it seems likely that such receptors contribute to the GABA_A_ receptor-mediated fraction of *g*_Cl_ and [Cl^–^]_i_ recovery. The novel method used here to estimate resting *g*_Cl_ could easily be applied also to many other preparations. It may be noted that manipulating Cl^–^ concentrations in the external solution may in many cases provide a useful alternative to loading cells with Cl^–^, as done here.

A significant contribution of glycine receptors to the resting *g*_Cl_ seems unlikely for several reasons. First, the competitive glycine-receptor blocker strychnine did not affect [Cl^–^]_i_ recovery (Fig. 7*A*). Second, in contrast to some GABA_A_ receptors, glycine receptors do not show any significant degree of spontaneous openings in the absence of ligand (Twyman and Macdonald, 1991; Beato et al., 2002; Bode et al., 2013).

Although ClC-2 channels seem to play a large role for the background conductance in CA1 pyramidal cells (Rinke et al., 2010), we did not find evidence for such channels in MPN neurons. Thus, it is unlikely that they contribute to resting *g*_Cl_ in these neurons. In cells where ClC-2 channels are present, their contribution to *g*_Cl_ may be expected to enhance the impact of conductive [Cl^–^]_i_ recovery even beyond that described in the present work. This may be of particular importance if the ClC-2-containing neurons show a limited transporter-dependent recovery capacity, such as indicated by our recordings from hippocampal neurons in the present study.

### Role of minis and neurosteroids in Cl^–^ homeostasis

Spontaneous neurotransmitter release may possibly play a special role in the control of conductive [Cl^–^]_i_ changes. The small postsynaptic currents generated do most often not induce major voltage fluctuations (Miles and Wong, 1986; Sayer et al., 1989; Rotaru et al., 2015). Accordingly, our results showed that, in control conditions, spontaneously released GABA does not significantly affect the recovery of [Cl^–^]_i_ after a high load. However, postsynaptic currents generated by GABA release onto MPN neurons may be prolonged by the action of the endogenous neurosteroid allopregnanolone (Haage and Johansson, 1999). We here showed that in the presence of a concentration (20 nM) that has been detected *in vivo* when animals are exposed to stress (Purdy et al., 1991), allopregnanolone significantly enhances the speed of [Cl^–^]_i_ recovery. Thus, the neurosteroid has the capacity to control [Cl^–^]_i_ dynamics in a situation when raised [Cl^–^]_i_ seems particularly likely, as a result of stress-induced enhancement of neural activity. Although a stress-induced reduction of inhibition may be important for, e.g., corticotrophin-releasing hormone neurons (Hewitt et al., 2009), it seems likely that other neurons may need a maintained functional inhibition during stress and that allopregnanolone may facilitate this via enhanced conductive [Cl^–^]_i_ recovery. Similarly, neurosteroid-induced enhancement of [Cl^–^]_i_ recovery may likely partly compensate for low levels of KCC2. However, as shown in our computations (Fig. 8*C*), in such situations there will be a trade-off between the rate of recovery and the minimum steady-state [Cl^–^]_i_.

The observed effect of 20 nM allopregnanolone on [Cl^–^]_i_ recovery can be ascribed to the postsynaptic currents (mIPSCs) generated by spontaneously released GABA, since baseline current was not affected. At micromolar concentrations, allopregnanolone directly activates GABA_A_ receptors as well as potentiates mIPSC frequency and prolongs mIPSC decay in MPN neurons (Haage and Johansson, 1999). In other cells, direct activation of GABA_A_ receptors may occur at 100 nM allopregnanolone (Shu et al., 2004), a concentration that may be reached during pregnancy (Nguyen et al., 2003), suggesting that steroid modulation of [Cl^–^]_i_ homeostasis via resting *g*_Cl_ through direct GABA_A_ receptor activation may be expected under physiologic conditions. Neurosteroid-controlled regulation of Cl^–^ homeostasis via the channels opened by spontaneously released GABA may possibly provide a novel functional role for spontaneous neurotransmission.

## Synthesis

Reviewing Editor: Alfonso Araque, University of Minnesota Decisions are customarily a result of the Reviewing Editor and the peer reviewers coming together and discussing their recommendations until a consensus is reached. When revisions are invited, a fact-based synthesis statement explaining their decision and outlining what is needed to prepare a revision will be listed below. The following reviewer(s) agreed to reveal their identity: Charles Zorumski, Andrea Barberis

Both reviewers are postitive about the manuscript and largely satisfied with the responses to the criticisms about the previous version reviewed in J Neurosci.

However, they have some concerns that need to be addressed and that I believe will enhance the strength of the manuscript.

I am looking forward to receive the revised version of the manuscript.

Specific comments of the reviewers:

Reviewer 1.

General comment:

The role of GABA receptors in the resting conductance of neurons is an understudied topic of importance in understanding factors that regulate basal neuronal excitability. The authors present a systematic study of the role of these receptors with the development of a model that could help other investigators.

Specific comments:

The authors have made a good faith effort to respond to the prior reviews, including new experimental data. My comments are minor and are mostly for clarification.

1. In the Abstract (line 28), the authors should include glycine receptors among the channels examined.

2. Introduction (line 60). ...input should be inputs.

3. Page 17. The authors comment about the percent of resting conductance accounted for by GABA receptors. They should do the same for glycine since they make a point of using both GABA and glycine to activate chloride currents in their other analyses.

4. Line 479. The term “spontaneously” open receptors has different meanings for different physiologists. In some cases this can refer to unliganded receptors. I suggest they delete the word “spontaneously” (this doesn't change their meaning and avoids the confusion).

5. Figure 2 legend, line 888. Panel C does not show voltage ramps.

Reviewer 2.

General comment:

The manuscript “Contribution of Resting Conductance, GABAA-Receptor Mediated Miniature Synaptic Currents and Neurosteroid to Chloride Homeostasis in Central Neurons” by Yelhekar and coworkers investigates the mechanisms regulating the intracellular chloride concentration in neurons. In particular this work aims at understanding the pathways of chloride extrusion after “massive chloride loading”, a condition that might approximate sustained and repetitive inhibitory synaptic activity.

By using electrophysiological and pharmacological approaches, the Authors conclude that chloride extrusion does not only occur through the conventional KCC2-mediated pathway, but it is significantly mediated by a chloride ions flux through GABAA receptors (at membrane resting potentials).

This topic is extremely relevant. Indeed, after inhibitory activity, the increase of intracellular chloride relaxes back to baseline in tens (or hundreds) of seconds, a time that is orders of magnitude longer than the synaptic integration times. This implies that the dynamics of chloride extrusion heavily impact on inhibitory short term synaptic plasticity with obvious consequences on the network functioning.

The present study is well conducted technically very sound. The experimental evidence shown, in general, supports the conclusion made by the Authors and increases our knowledge about the mechanisms of chloride homeostasis during inhibitory activity. Indeed, as far as I know, it is currently known that in addition to KCC2, only ClC-2 channels may contribute to “assist” chloride extrusion.

However, there are some points that need to be clarified.

Major points:

Although the Authors convinced me that GABAA receptors in mediating chloride extrusion, the experiments carried out to dissect out the type of GABAA receptors (tonic vs phasic) are not fully conclusive.

1. While PTX is reported to slow the rate of [Cl-] recovery, Gabazine is ineffective. Basing on these experiments the authors conclude that the chloride extrusion is mediated by Gabazine-independent receptors spontaneously open. Similar receptor pharmacology has been described in Wlodarczyk et al., (2013). In this study, however, such “spontaneously open receptors” have been shown not to contain the delta subunit. In the experiment with the positive allosteric modulator DS2, in contrast, the Authors state: “[Cl-]i recovery may be pharmacologically enhanced via positive modulation of δ-subunit containing GABAA receptors”. How the authors reconcile this discrepancy also in the light that delta-containing GABAA receptors have been extensively shown to be blocked by Gabazine? Which GABAAR subunit composition do the authors believe is mediating such [Cl-] recovery process?

2. It is a bit puzzling to me to understand why the authors used Allopregnanolone to dissect out the contribution of synaptic GABAA receptors (mediating mIPSCs). Indeed, this neurosteroid is mainly known for its effect on delta-containing, tonic, extrasynaptic GABAA receptors. By using this drug, it is difficult to specifically isolate the component mediated by synaptic receptors. Why the authors did not use benzodiazepines (BZDs) instead? BZDs selectively potentiate gamma-containing synaptic receptors (mediating mIPSCS) and have been reported to slow down the decay kinetics of mIPSCs.

3. If the authors think that receptors openings during synaptic activity (mIPSCs) are involved in the chloride recovery, it would be very elegant to selectively increase mIPSCs frequency by using, for instance, latrotoxin or by playing with the Ca/Mg ratio. In this case the effect of postsynaptic receptor would be unmasked without the need to interfere with the receptors themselves.

4. It is surprising that the [Cl-]i recovery does not change in the presence of TTX, since the frequency of sIPSCs is typically higher than that of mIPSCs. However in some preparations the action potential (AP) driven activity is similar to AP-independent activity. Is that the case of your experimental model (isolated preoptic neurons)? Please comment on this.

Minor points

1. In the abstract and in several other points it is stated that AP-independent currents (mIPSCs) are important for chloride recovery. Why is it important to specify “impulse-independent release of GABA”? Wouldn't the rationale of the study hold true if the current were AP-driven (sIPSCs)? After all, in the brain most of IPSCs are AP driven.

2. I would change “impulse” with “action potential”

3. I found a bit distracting the use of sentences between brackets after a period. Example: “......recovery of a low [Cl-]i. (In the absence of Cl- transporters, the Cl- equilibrium potential, ECl, will settle at the membrane resting potential.)”. I would get rid of brackets and link the sentence to the previous.

4. I found the discussion a bit verbose
